# Health Maintenance Organization–mHealth Versus Face-to-Face Interaction for Health Care in Israel: Cross-Sectional Web-Based Survey Study

**DOI:** 10.2196/55350

**Published:** 2024-09-30

**Authors:** Avi Zigdon, Moti Zwilling, Ofek Zigdon, Orna Reges

**Affiliations:** 1 Department of Health Systems Management School of Health Sciences Ariel University Ariel Israel; 2 Department of Economics and Business Administration Ariel University Ariel Israel; 3 Faculty of Medicine The Hebrew University of Jerusalem Jerusalem Israel

**Keywords:** HMO-mHealth, mHealth, face-to-face, digital health, digital health apps, eHealth, HMO-mHealth adoption, health care, mHealth adoption, mobile phone, HMO, health maintenance organization

## Abstract

**Background:**

Health maintenance organization–mobile health (HMO-mHealth) services have a direct impact on patients’ daily lives, and HMOs regularly expand their range of mHealth services. HMO-mHealth apps are saving HMOs time and money, as services are becoming more accessible to patients. However, the willingness to use mHealth apps depends on user perception. Although mHealth apps can change the relationship dynamic between HMOs and patients, patients prefer to use them to facilitate face-to-face interactions rather than replace them.

**Objective:**

This study aims to examine the extent to which Israeli adults prefer adopting health care services using HMO-mHealth as a replacement for face-to-face interaction.

**Methods:**

Israeli adults aged ≥18 years completed an electronic questionnaire. Data were collected from December 2020 to February 2021. All services in the main HMO-mHealth apps of the 4 Israeli HMOs were mapped. The 29 health care services used in this study were identical in all 4 HMO-mHealth apps in Israel. The association between sociodemographic characteristics and health condition with preference for HMO-mHealth or face-to-face interaction was analyzed separately for each health service by using a logistic model.

**Results:**

A total of 6321 respondents completed the questionnaire (female: 4296/6321, 68%; male: 2025/6321, 32%). Approximately 80.9% (5115/6321) to 88.2% (5578/6321) of the respondents preferred using HMO-mHealth apps for administrative matters. However, 55.3% (3498/6321), 52.2% (3301/6321), and 46.9% (2969/6321) preferred face-to-face meetings for the initial medical diagnosis, medical treatment, and medical diagnosis results, respectively. Seven main variables were found to be associated with HMO-mHealth adoption, including gender, age, education, marital status, religious affiliation, and subjective health condition. Female respondents were more likely than male respondents to prefer HMO-mHealth apps for administrative matters and face-to-face interaction for personal medical diagnosis and treatment (odds ratio [OR] 0.74, 95% CI 0.67-0.83; *P*<.001 and OR 0.82, 95% CI 0.74-0.92; *P*<.001, respectively). Married individuals preferred using HMO-mHealth apps over face-to-face meetings for a new medical diagnosis (OR 1.31, 95% CI 1.15-1.49; *P*<.001) or treatment (OR 1.34, 95% CI 1.18-1.52; *P*<.001). Improved health perception was associated with higher preference for HMO-mHealth apps across all health care services in this study (OR 1.11, 95% CI 1.02-1.22; *P*<.02 to OR 1.38, 95% CI 1.25-1.53; *P*<.001). No significant association was found between the presence of a chronic disease and the preferred mode of interaction for most services.

**Conclusions:**

HMO-mHealth is proving to be a robust and efficient tool for health care service delivery. However, there are barriers that affect vulnerable populations when adopting HMO-mHealth. Therefore, it is important to tailor HMO-mHealth apps for older adults, the chronically ill, and minorities in society, as these groups have a greater need for these services. Future studies should focus on identifying the barriers that affect the utilization of HMO-mHealth in these groups.

## Introduction

The rapid development of mobile health (mHealth) directly impacts patients’ daily lives, relationships, and communication with health maintenance organizations (HMOs). In the absence of an epidemic and its consequences, the use of mHealth apps is at the discretion of the patient [1,2], and only a few use them frequently [3,4]. Typically, mHealth apps are used to independently manage medical interventions and personalize treatments, aiming to reduce the demand on health care providers and eliminate geographic barriers [5]. Nevertheless, the potential of mHealth is vast [6]. The medical information and services provided to patients by HMOs can be personalized to their specific needs anytime and anywhere [7,8]. Utilization of mHealth is beneficial in many ways. It enables self-management of disease, evaluation of personal medical information, and easier and more accessible contact with medical professionals [9-13]. mHealth can also help support patients’ lifestyles [13], improve patients’ health status [14], improve their quality of life and health outcomes, and reduce the incidence of disease [12]. Sometimes, they even help with hospitalization [2] and take over the roles of general practitioners [15]. In addition, mHealth influences patients’ sense of autonomy and their need for well-being [2].

HMOs are regularly expanding the services offered in mHealth. The use of information and communication technologies to meet the needs of health systems around the world is increasing [16]. In England, for example, the National Health Service has developed mHealth apps that allow users to make appointments with doctors, request prescription renewals and order medications, receive medical advice, view personal medical records, declare their willingness to donate organs, and with the COVID-19 pandemic outbreak, obtain authorizations and information for outbreak durations [17].

In Germany, the Digital Healthcare Act, which was introduced in 2019, allows health insurers to promote the development of apps that include the ability to renew a prescription, provide medical advice via video calls, and access a secure data network from anywhere in the country. In addition, the use of mHealth apps can help promote digital health literacy among patients to achieve equal access and high involvement on the patients’ part [18]. Like the health systems in England and Germany, that in Israel is primarily a public health system. According to the Israeli health insurance law [19], every resident in Israel is entitled to health insurance that includes a standard basket of services, which is termed the Basic Healthcare Basket, established by law. In addition to this system, residents can expand the basic services basket by purchasing an additional basket of services from HMOs. Health care services are provided to all Israeli residents by 4 health care providers (Clalit Health Services, Maccabi Healthcare Services, Leumit Health Services, and Kupat Holim Meuhedet) to which the residents are registered [19]. In May 2011, mHealth apps launched by 2 HMO funds were launched: Maccabi Healthcare Services [20] and Leumit Health Services [21]. In August 2011, the mHealth app of Kupat Holim Meuhedet was launched, and in February 2012, the mHealth app of Clalit Health Services was launched [22,23]. Over the years, the services offered by HMOs through mHealth apps have evolved. Currently, they offer a wide range of services both in the main health care services app and in additional apps (eg, childcare support, pregnancy support). During the COVID-19 pandemic, the use of telehealth services increased [24], and there was an increase in the use of HMO mHealth services, especially among patients with chronic diseases [25]. There was also an increase in the use of COVID-19 management apps (monitoring exposure to diagnosed patients and diagnosing symptoms) [24]. The use of remote medicine services has also increased in Israel [26,27], wherein some of these health care services were provided by the HMO-mHealth apps [20-23]. However, the willingness to use mHealth apps is influenced by the user’s perception of perceived usefulness, perceived ease of use, subjective norms [28], app quality, and user health literacy [29,30]. mHealth apps are promising platforms that can be used to provide efficient and convenient access to therapeutic services [31]. The use of mobile platforms is highly recommended in combination with face-to-face interventions to support people with their daily routines [6]. However, mHealth appointments were found to be more expensive than face-to-face health care appointments [32]. A meta-ethnographic review of qualitative studies by Vo et al [6] sheds light on the dynamics of patient engagement and opportunities to help patients become more empowered through the use of mHealth apps. Vo et al [6] reviewed 43 papers on qualitative studies that addressed patient evaluation, expectations, and perceptions of mHealth apps. Patients described mHealth apps as tools that enable discussions with their health care providers that can improve adherence to care and their health care experience. mHealth apps were described as a tool for patients to engage in a 2-way dialogue. Although mHealth apps can change the dynamics of patient-provider relationships by providing relevant information for conducting assessments, diagnoses, registering for treatments, etc, patients prefer to use them simply as a tool to facilitate rather than replace these relationships [6]. The aim of this study was to examine the extent to which adults in Israel prefer to adopt HMO-mHealth apps and receive health care services via HMO-mHealth as a substitute for face-to-face interaction and to identify factors associated with these preferences.

## Methods

### Study Design

We designed a cross-sectional web-based survey to examine the preferences of adults in Israel for adopting HMO-mHealth apps and receiving health care services versus HMO-mHealth as a substitute for face-to-face interaction. Data for this study were collected from December 2020 to February 2021. Respondents were not required to provide any identifying information, and any participant could stop the survey at any point.

### Participant Recruitment

The questionnaire was designed on the Qualtrics platform and distributed by research assistants via social media (WhatsApp groups, Facebook, etc). Our target population was Israeli adults aged ≥18 years, who were sampled using convenience sampling. Responders were allowed to fill in the questionnaire electronically only once. To estimate the required sample size, we relied on data from the Central Bureau of Statistics in Israel. The population of the State of Israel in January 2024 was estimated to be around 9.855 million [33]. Since the proportion of mHealth use in the population is unknown and is assumed to be a maximum of 50%, with a significance level of 5%, a sample size of 384 residents was calculated for this study. In practice, there was a high response rate, and 6321 Israeli adults completed the research questionnaire. It was decided to use all the data to obtain greater validity.

### Survey Development and Definition of Variables

All services available in the main HMO-mHealth apps of the 4 Israeli HMOs were mapped. A total of 29 health care services, which were identical across all 4 HMO-mHealth apps in Israel, were selected for this study and included in the HMO-mHealth/face-to-face interaction preference questionnaire. These services included administrative matters such as setting a visit or changing a visit date as well as medical services such as receiving a medical diagnosis or treatment. For each of the 29 items, participants were asked whether they preferred mHealth or face-to-face interaction to receive the health service. Each item was tested as a separate outcome. In addition, the following information was self-reported by the participants: gender, age (years), marital status (married/single/divorced/widowed), number of children, birthplace (Israel/other), religion (Jewish/non-Jewish), religiosity (secular/religious), education (uneducated/elementary-middle school/high school/vocational training/BA/MA/PhD), residence (Center, North, or South Jerusalem), subjective health condition (from 1: worst to 5: very good), and previous diagnosis of chronic illness by a licensed physician (yes/no).

### Data Analysis

The main characteristics of the study population were described using percentages for categorical variables and means with standard deviations for continuous variables. Chi-square tests or independent-sample 2-sided *t* tests were employed to evaluate the association of categorical or continuous predictors, respectively, with each of the 29 health services included in this study. Variables that were significant (*P*<.05) in the univariable tests for at least one of the health care services were included in the final multivariable logistic models. The association between significant personal characteristics with the preference of mHealth or face-to-face interaction was assessed separately for each health care service by using multivariable logistic regression analysis. In each model, a particular health service was evaluated as the dependent variable (0=face-to-face; 1=mHealth). The predictor variables were entered simultaneously into each model and included sociodemographic characteristics (gender, age, marital status, birthplace, number of children, education, religion, religiosity, residence), subjective health condition, and previous diagnosis of chronic illness. To mitigate the potential for spurious statistical significance arising from multiple tests, Benjamini-Hochberg adjusted *P* values were computed using WinPepi version 11.65 [34]. *P* values less than .05 were considered statistically significant.

### Ethics Approval

This study was approved by the ethics committee of Ariel University (AU-HEA-AZ-20201217). All participants were informed of the aims of the study in an introductory section, and they gave their informed consent to participate in the study. No conditioning questions were asked to conduct the survey, as health care services are provided to all residents of Israel by 4 health care providers to which the residents are registered.

## Results

Between December 2020 and February 2021, 6321 Israeli adults completed the study questionnaire. The main characteristics of the study population are described in Table 1. The mean age of the participants was 35.42 (SD 13.09) years, and the majority were female (4296/6321, 68%), Jewish (5234/6321, 82.8%), and born in Israel (5283/6321, 83.6%). About half were married (3299/6321, 52.2%), lived in the center of Israel (3104/6321, 49.1%), had no children (3253/6321, 51.5%), and had an academic degree (BA, MA, or PhD: 3152/6321, 49.9%). More than half described themselves as secular or traditional (3546/6321, 56.1%). Most participants had no chronic diseases (5235/6321, 82.8%) and declared that they were in very good health condition (3775/6321, 59.7%).

**Table 1:** General characteristics of the participants (N=6321).

Demographic variables			Values
**Gender, n (%)**			
	Female	4296 (68)	
	Male	2025 (32)	
**Age (years)**			
	≤39, n (%)	4383 (69.3)	
	40-64, n (%)	1723 (27.3)	
	65-74, n (%)	164 (2.6)	
	≥75, n (%)	51 (0.8)	
	Mean (SD)	35.4 (13.1)	
**Marital status, n (%)**			
	Married	3299 (52.2)	
	Single	2708 (42.8)	
	Divorced/widowed	313 (5)	
**Children, n (%)**			
	0	3253 (51.5)	
	1	557 (8.8)	
	2	803 (12.7)	
	3	787 (12.4)	
	4	493 (7.8)	
	5	219 (3.5)	
	≥6	209 (3.3)	
**Birthplace**, **n (%)**			
	Israel	5283 (83.6)	
	Other	1038 (16.4)	
**Religious affiliation**, **n (%)**			
	Jewish	5234 (82.8)	
	Non-Jewish	1087 (17.2)	
**Religiosity**, **n (%)**			
	Secular	3546 (56.1)	
	Religious	2775 (43.9)	
**Education,** **n (%)**			
	Uneducated	50 (0.8)	
	Elementary/middle school	135 (2.1)	
	High school	2245 (35.5)	
	Vocational training	739 (11.7)	
	Bachelor of Arts	2385 (37.7)	
	Master of Arts/Doctor of Philosophy	767 (12.2)	
**Subjective health condition** **,** **n (%)**			
	Very good	3775 (59.7)	
	Good	2184 (34.6)	
	Moderate	299 (4.7)	
	Not good	54 (0.9)	
	Bad	9 (0.1)	
**Chronic illness**, **n (%)**			
	Yes	1086 (17.2)	
	No	5235 (82.8)	

**Figure 1:**
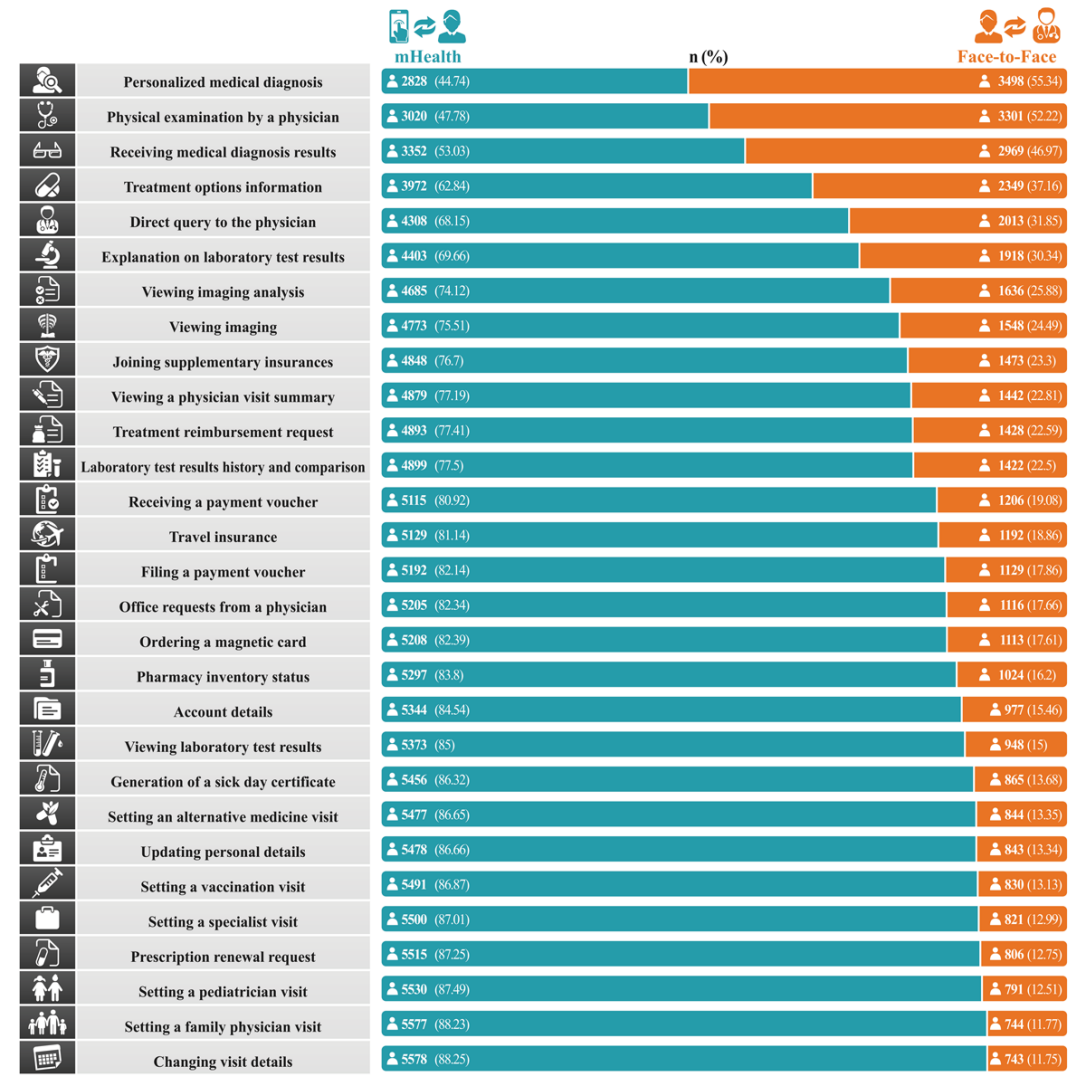
Participants’ preferences for receiving health care services via health maintenance organization–mobile health apps or face-to-face interaction for 29 health services included in this study. mHealth: mobile health.

Figure 1 shows participants’ preferences for receiving health care services via HMO-mHealth apps or face-to-face interaction for 29 health services offered by all HMO-mHealth apps in Israel. The vast majority of the participants (5115/6321, 80.9% to 5578/6321, 88.2%) preferred to use HMO-mHealth apps for administrative services such as changing visit dates, scheduling a visit for vaccination, submitting a prescription renewal request, and generation of a sick-day certificate. When the primary purpose of the visit was for medical services involving treatment or diagnostics, more individuals preferred face-to-face meetings over HMO-mHealth services. Specifically, 55.3% (3498/6321) of the participants reported that they preferred a face-to-face meeting to receive the initial medical diagnosis, and 52.2% (3301/6321) reported that they preferred a face-to-face meeting to receive medical treatment. Approximately 46.9% (2969/6321) preferred a face-to-face meeting for receiving medical diagnosis results.

**Figure 2:**
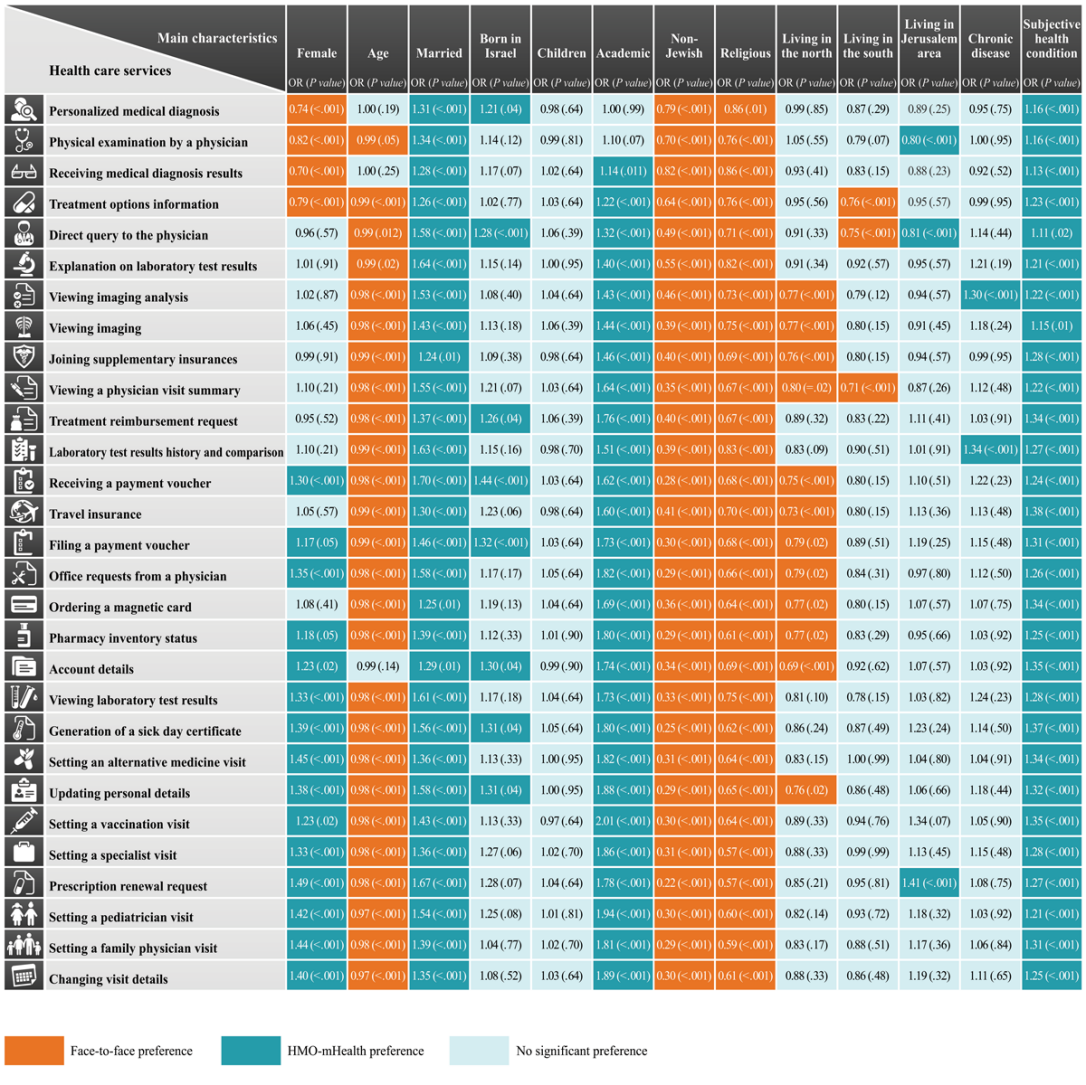
Multivariable logistic regression models of the factors associated with the adoption of health maintenance organization–mobile health versus face-to-face interaction (please also refer to Multimedia Appendix 1). HMO: health maintenance organization; mHealth: mobile health; OR: odds ratio.

Figure 2 summarizes the association of personal characteristics with the preference for mHealth or face-to-face interaction. The multivariable logistic regression models revealed differences in the factors associated with preferences for use of health services via HMO-mHealth apps or face-to-face interaction. Among sociodemographic characteristics, sex, age, marital status, education level, ethnicity, and religiosity were significantly associated with the preference of mHealth/face-to-face interaction for most health care services. Specifically, compared to unmarried individuals, married individuals preferred using HMO-mHealth apps over face-to-face meetings to obtain a new medical diagnosis (odds ratio [OR] 1.31, 95% CI 1.15-1.49; *P*<.001) or treatment (OR 1.34, 95% CI 1.18-1.52; *P*<.001) and for changing visit details (OR 1.35, 95% CI 1.10-1.66; *P*=.004) or scheduling a new visit with a primary care physician (OR 1.39, 95% CI 1.13-1.71; *P*<.001), pediatrician (OR 1.54, 95% CI 1.26-1.89; *P*<.001), or specialist (OR 1.36, 95% CI 1.12-1.66; *P*<.001). Individuals with an academic background were more likely to prefer HMO-mHealth apps over face-to-face meetings when administrative issues were involved, such as making an appointment with the family physician (OR 1.81, 95% CI 1.53-2.14; *P*<.001) or changing visit details (OR 1.89, 95% CI 1.60-2.24; *P*<.001) and for medical information such as direct query for the physician (OR 1.32, 95% CI 1.18-2.48; *P*<.001), viewing imaging analysis (OR 1.43, 95% CI 1.27-1.61; *P*<.001), or a visit summary (OR 1.64, 95% CI 1.45-1.87; *P*<.001). However, no association was found between academic status and preferred mode of interaction for medical diagnosis and treatment. Non-Jewish and religious individuals were less likely to use HMO-mHealth apps and preferred face-to-face interaction for both administrative and medical purposes. Female respondents were more likely than male respondents to prefer HMO-mHealth apps for administrative services and face-to-face interaction for personal medical diagnosis or treatment (OR 0.74, 95% CI 0.67-0.83; *P*<.001 and OR 0.82, 95% CI 0.74-0.92; *P*<.001, respectively).

A better subjective perception of health condition was associated with a higher preference for using HMO-mHealth apps. However, no significant association was found between the presence of chronic disease and the preferred mode of interaction for most services.

## Discussion

### Principal Findings

Adoption of mHealth by patients is a complex and multidimensional process with its own advantages, disadvantages, and barriers. This study was the first to examine the extent to which adults in Israel prefer to receive health care services through HMO-mHealth as a substitute for face-to-face interaction and to identify factors associated with these preferences. Approximately 46.9% (2969/6321) of our study participants preferred face-to-face interaction for the initial treatment or diagnosis rather than through HMO-mHealth apps (represented by the items, namely, personalized medical diagnosis, physical examination by a physician, and receiving medical diagnosis results). However, 80.9% (5115/6321) to 88.2% (5578/6321) of our study participants were interested in receiving administrative services through HMO-mHealth apps (demonstrated in items such as changing visit details, setting a family physician visit, and prescription renewal request). Nevertheless, 11.7% (743/6321) to 19.1% (1206/6321) of the participants were still interested in receiving administrative and information services through face-to-face meetings. Our axiom is that the respondents have adequate digital literacy since the questionnaire was distributed and filled out using a digital tool. However, good digital literacy is not necessarily a guarantee of the ability to use digital health services [1], and the validity of information in mHealth apps that focus on emotional content may influence the adoption of HMO-mHealth services, even among those with high digital literacy [6,35]. The high usage rate of HMO-mHealth in this study can be explained by the Unified Theory of Acceptance and Use of Technology (UTAUT), which states that effort expectancy (defined as the degree of ease of use) affects the acceptance and use of HMO-mHealth [36]. Seven main variables were found to be associated with HMO-mHealth adoption, including gender, age, education, marital status, religious affiliation, and subjective health condition.

Our results show that women are more likely to adopt HMO-mHealth apps for medical information or administrative services (eg, prescription renewal request, setting a family physician visit, account details inquiry). However, they prefer face-to-face interactions with physicians for receiving medical services with treatment or diagnostic significance (eg, personal medical diagnosis, medical examination, receiving the results of medical diagnoses, information on treatment options). This result is interesting because one might assume that gender has a similar effect on the adoption of all HMO-mHealth services and that there are no differences between the different types of services. Previous studies suggest that there are generally no clear trends in gender differences in the willingness to adopt mHealth technologies [3,14,25,27,36-38]; gender is mentioned as a moderating variable in mHealth studies dealing with UTAUT, and it plays a moderating role with threat appraisal and coping appraisal factors in mHealth adoption behavior [39,40]. However, traditional face-to-face health care is frequently preferred over digital health care [1], and women, in particular, tend to be more skeptical about relying solely on mHealth for treatment and place higher value on in-person doctor visits [41]. This inclination is likely influenced by greater health anxiety and risk aversion among women [42-45].

Consistent with previous studies [11,14,27,37], our study also shows that age, education, religious affiliation, and ethnicity were barriers to HMO-mHealth adoption. On the one hand, people with a higher level of education and younger individuals had a higher tendency to adopt HMO-mHealth services compared to older individuals and those with a lower level of education who preferred face-to-face meetings. On the other hand, societal minorities (non-Jews) and religious individuals tended to prefer face-to-face meetings over the adoption of HMO-mHealth services. It is possible that internet use is perceived as more accessible among young people, and those with higher education are perceived as having better digital literacy [4,46]. However, our study shows that people with a higher level of education and younger individuals who have a poor subjective health condition generally prefer face-to-face meetings. Based on this finding, it can be assumed that despite the effectiveness of HMO-mHealth services, young and educated people still find it necessary to have human contact with a health provider for diagnosis and treatment.

This study also shows that ethnic minorities (non-Jewish) and religious people would prefer face-to-face meetings over HMO-mHealth for all health care provider services examined in this study. There is evidence of the effectiveness of internet-based interventions that improve the health of ethnic minorities [47]. These ethnic minority groups reported greater interest in using mHealth technologies than the nonminority population [48]. A recent study found that there are significant ethnic differences in the use of telemedicine between different ethnic groups [27]. However, the association of religion with the adoption of health technologies has, to our knowledge, not yet been sufficiently investigated. The adoption of technologies by religious people is considered at higher-than-average risk, and previous studies have shown that religious leaders can influence the opinions and behaviors of religious people when adopting medical technologies [49]. We link these 2 facts because we believe that barriers to HMO-mHealth adoption can be associated with ethnicity and religion. Both cultural and community characteristics, language barriers, emotional state, and health and digital literacy can be assumed to play an important role in HMO-mHealth adoption [46,50]. A study examining the influence of cultural aspects on the adoption of mHealth in 3 different countries, based on UTAUT, found that cultural differences have a decisive effect on the adoption behavior model and concluded that mHealth services must be adapted to the culture of the population for which the services are developed [51].

Greater involvement of minorities in the development of HMO-mHealth will lead to the adoption of these tools by minorities [52], and the involvement of leaders from these communities in the development of technological medical products will enable broader adoption of HMO-mHealth in these populations. Another interesting finding of this study is that married people would prefer to adopt HMO-mHealth over face-to-face meetings. A systematic literature review [53] and narrative synthesis showed that the effects of marital status on mHealth uptake are inconsistent across studies. Some studies suggest that marital status has no significant effect on the use of mHealth services, while others propose possible differences in the intention to use depending on marital status [53]. In our opinion, addressing family health needs and marital responsibilities can increase interest in technologies that improve health monitoring and save time. Since there is a gender dynamic in most marital relationships regarding domestic responsibilities and health care decisions, this is likely to have an impact on the adoption of HMO-mHealth.

This study raises an interesting question: for whom are HMO-mHealth services really intended for? In this study, it was found that patients with chronic diseases do not prefer to use HMO-mHealth services. Conversely, people who describe their subjective health as good are more likely to use HMO-mHealth services. It is generally assumed that patients with chronic diseases will adopt more mHealth services [54], as mHealth is seen as part of medical practice and a factor that can support patients with chronic diseases [37]. There is evidence in the literature that subjective health condition is a factor that influences the use of digital means to access health care services [55]. However, studies show that the presence of a chronic disease does not predict demand for medical services [54,56-58]. It can be assumed that the support provided by the family of a patient with chronic diseases will reduce the need for HMO-mHealth services [58].

### Limitations

Our study has several limitations. First, due to the cross-sectional nature of our study, it is not possible to establish causation or demonstrate cause-and-effect relationships. Second, the data were self-reported. The researchers were not able to assess whether the questionnaire was completed during work or leisure time or whether health limitations influenced the completion of the questionnaire. Third, the services in the HMO-mHealth apps are similar, but the interaction between the patient and the health care provider in each app may be different. It is possible that a better response from the health care provider for certain services in the HMO-mHealth apps could influence the information collected in the questionnaire. Fourth, health literacy was not examined in this study. It is likely that the level of health literacy may also influence patients’ interactions with HMO-mHealth services.

### Conclusions and Future Directions

HMO-mHealth proves to be a robust and efficient tool for health care service delivery when compared to face-to-face health care interaction. However, barriers that affect vulnerable populations in HMO-mHealth adoption still exist. HMO-mHealth services will not be able to completely replace face-to-face interactions with health care providers and will be a complementary tool for face-to-face meetings with therapists. The utilization of health applications within health services constitutes an increasingly substantial component of the communication interface between the health care system and its patients, accompanied by a rise in the array of services offered through HMO-mHealth. On the one hand, this study’s outcomes furnish insights for policy makers engaged in the development of HMO-mHealth services, facilitating the formulation of culturally sensitive HMO-mHealth services. On the other hand, there exists a compelling necessity for policy makers to institute a comprehensive training regimen aimed at equipping patients with the requisite skills for utilizing HMO-mHealth. This is particularly imperative for aiding demographic segments such as the older adults, chronically ill patients, and societal minorities, who are in greater need of these services. Health care providers need to develop intervention plans for accessibility, adaptation, and implementation of HMO-mHealth in this population. Such adaptations are essential for mitigating health care inequalities in Western societies. To provide an optimal response to these populations, future studies should focus on identifying the barriers that affect the utilization of HMO-mHealth in these groups. Future studies should also use longitudinal designs to better establish cause-and-effect relationships between variables; assess health literacy, which is a potential factor in the adoption of HMO-mHealth; and include comparisons with mHealth adoption in other countries. These future studies could significantly enhance our understanding of the cultural and systemic variations.

## Appendix

Multimedia Appendix 1 Supplementary data.

## Abbreviations

HMO: health maintenance organization
mHealth: mobile health
OR: odds ratio
UTAUT: Unified Theory of Acceptance and Use of Technology

## Data Availability

The data sets generated and analyzed during this study are available from the corresponding author upon reasonable request, subject to approval by the ethics committee of Ariel University.
